# Improving the Robustness of Time Difference of Arrival Estimation Based on the Energy Center of Gravity Rearrangement

**DOI:** 10.3390/s23249720

**Published:** 2023-12-09

**Authors:** Peng Zhang, Hongyuan Wen, Zhiyong Xu, Zhao Zhao

**Affiliations:** 1 School of Electronic and Optical Engineering, Nanjing University of Science and Technology, Nanjing 210094, China; zhangpengice@njust.edu.cn (P.Z.); zhaozhao@njust.edu.cn (Z.Z.); 2 Taizhou Institute of Science and Technology, Nanjing University of Science and Technology, Taizhou 225300, China; 22009025@nustti.edu.cn

**Keywords:** communication, dual-channel, energy aggregation, localization, time-delay estimation, time difference rearrangement synchrosqueezing transformation

## Abstract

An accurate estimation of the time difference of arrival (TDOA) is crucial in localization, communication, and navigation. However, a low signal-to-noise ratio (SNR) can decrease the reliability of the TDOA estimation result. Therefore, this study aims to improve the performance of the TDOA estimation of dual-channel sensors for single-sound sources in low-SNR environments. This study introduces the theory of time rearrangement synchrosqueezing transform (TRST) into the time difference of arrival estimation. While the background noise TF points show random time delays, the signal time-frequency (TF) points originating from uniform directions that exhibit identical lags are considered in this study. In addition, the time difference rearrangement synchrosqueezing transform (TDST) algorithm is developed to separate the signal from the background noise by exploiting its distinct time delay characteristics. The implementation process of the proposed algorithm includes four main steps. First, a rough estimation of the time delay is performed by calculating the partial derivative of the short-time cross-power spectrum. Second, a rearrangement operation is conducted to separate the TF points of the signal and noise. Third, the TF points on both sides of the time-delay energy ridge are extracted. Finally, a refined TDOA estimation is realized by applying the inverse Fourier transformation on the extracted TF points. Furthermore, a second-order-based time difference reassigned synchrosqueezing transform algorithm is proposed to improve the robustness of the TDOA estimation by enhancing the TF energy aggregation. The proposed algorithms are verified by simulations and experiments. The results show that the proposed algorithms are more robust and accurate than the existing algorithms.

## 1. Introduction

Time delay estimation (TDE) is crucial in many fields, including radar, navigation, sonar, seismic monitoring, acoustic source localization, environmental monitoring, and space exploration applications [1,2,3,4,5,6]. Accurate TDE is crucial for source localization systems, such as wireless communication and instantaneous finding systems, which track the relative location of a goal [7,8,9,10]. There are two types of TDE distinguished by their active and passive detection methods, namely the time of arrival (TOA) estimation and the time difference of arrival (TDOA) estimation. The former estimates the lag between a signal’s emission and its echo’s reception. The TOA estimation has mainly been used in active systems, such as active sonars and wireless navigation systems. However, the TDOA estimation determines the variation in the wavefront travel time between distant receiving sensors in an array. Typically, it has been used in passive systems, such as passive radars and microphone arrays. Still, there are numerous challenges in TDOA estimation that must be addressed to meet the requirements for high accuracy and robustness of different applications [11,12,13,14]. In recent years, many TDOA algorithms have been proposed, each with strengths and weaknesses. The cross-correlation (CC) algorithm is a prominent TDOA technique, assuming that signals satisfy the stationarity criteria. This approach has been highly favored due to its efficient computational capabilities. The CC algorithm for the TDOA estimation requires statistical knowledge about the independence of the signal and noise. Despite having a low SNR, the CC algorithm still provides accurate estimates. However, suppose the instantaneous correlation energy of the effective signal is lower than that of the noise. In that case, the CC approach will incorrectly estimate the delay since it will attribute the delay to the noise instead of the effective signal [15].

To enhance the TDOA estimation efficiency in low-SNR environments, recent research has proposed the multi-channel cross-correlation coefficient (MCCC) technique. This technique uses the spatial redundancy of various sensors to decrease the noise’s influence on the TDE result. Compared to the CC technique, the MCCC technique is much more resilient to noise and can achieve significantly higher estimation accuracy under the same SNR conditions. However, in a reverberant environment, the MCCC technique’s performance can substantially decline [16]. In Ref. [17], multi-channel spatiotemporal sparse prediction (MCSTSP) and multi-channel spatiotemporal group sparse prediction (MCSTGSP) were proposed for the TED. By exploiting the sparsity of the spoken signal prediction parameter matrix, these algorithms can develop an optimization approach that incorporates the MCCC and MCSTP to improve the TDE performance. Compared to the MCCC, the MCSPSP and MCSTGSP are much more effective in handling reverberation. In Ref. [18], a frequency-sliding generalized cross-correlation (FS-GCC) method was proposed. This method extracts the phases of different frequency bands of the inter-correlation spectrum to construct the CC matrix. In addition, the singular value decomposition (SVD) and weight SVD methods are used to obtain robust TDEs from the cross-correlation matrix. In Ref. [19], an improved algorithm for multiple-signal classification (IP-MUSIC) was proposed. This algorithm uses a smoothing technique to express the CC result in the form of a covariance matrix, and the TDE is obtained by the MUSIC algorithm. Further, a technique for estimating time delays below the Rayleigh distance using the eigenvalue decomposition and a function called the exponential kernel correlation (EKC) was proposed. The non-Gaussian impulsive noise is considered to have less of an influence due to the EKC function. An advanced algorithm that combines the EKC function and the MUSIC algorithm was proposed to improve estimation accuracy in noisy environments. This algorithm projects the signal subspace to the noise subspace. However, it requires knowledge of the number of sources and may have reduced accuracy under the conditions of a low SNR or limited data [20]. An improved solution called the Dual-Path Transfer Function (DPTF) model was introduced to measure time delay in reflection environments, and a rapid DPTF algorithm that uses gradient-based techniques to obtain accurate values was developed. However, further improvements are still necessary to enhance the model’s robustness, especially when operating under the low-SNR conditions [21].

The aforementioned studies on the TDOA estimation have achieved good estimation results [22,23,24]. However, the TDE performance can be further improved in a low-SNR environment. In view of that, this study proposes an innovative TDOA algorithm aiming at non-stationary signals. In addition, the analysis in this paper is founded upon two hypotheses:

(1) After rearranging the short-time cross-power spectrum (STCPS) based on the delay characteristics, the TF points with stronger SNR are closer to the actual time-delay ridges;
(2) In each narrow frequency band, the noise power changes slowly over time but remains relatively constant overall.

The main contributions of this work are as follows:

(1) The theory of time rearrangement synchrosqueezing transform (TRST) is extended to the time difference of arrival estimation, and a time difference rearrangement synchrosqueezing transform algorithm is proposed to enhance the performance of the TDOA estimation by separating the signal from the background noise by exploiting the distinct time delay characteristics;
(2) A second-order-based time difference reassigned synchrosqueezing transform algorithm is developed to improve the robustness of the TDOA estimation by enhancing the TF energy aggregation.

The rest of this paper is structured as follows. Section 2 proposes two time difference reassignment transform algorithms. Section 3 introduces the evaluation of time difference reassigned synchrosqueezing transformation in the TDOA estimation. Finally, Section 4 presents the results and highlights the main achievements compared to the existing algorithms.

## 2. Proposed Algorithm

This section introduces two innovative TDOA estimation algorithms based on the rearrangement method. Specific details are provided in Section 2.1 and  Section 2.2.

**Figure 1:**
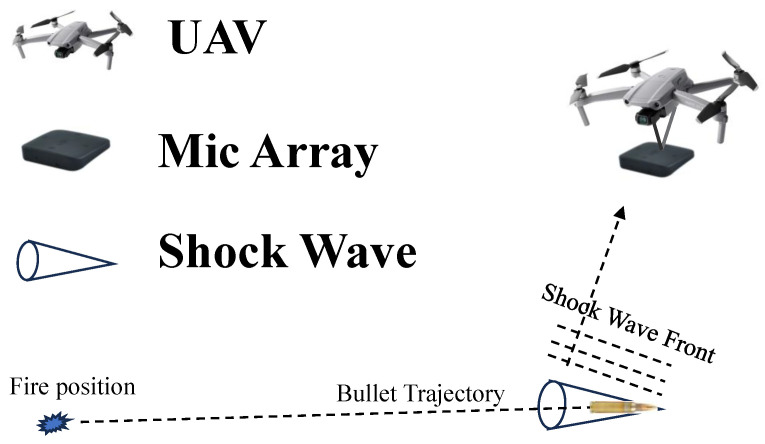
Illustration of the localization process of a shock wave source.

This paper presents the research on the time delay estimation of a shock wave. This research belongs to the study of firing point localization. The shock wave is an impulsive signal produced by a bullet squeezing the air as it travels through the air at supersonic speeds. The shock wave expands as a cone behind the bullet, having the wavefront propagating outward at the speed of sound. The TDE of the shock waves plays a crucial role in fire position localization systems. The application of the TDOA in the passive detection of a fire position through gunfire is shown in Figure 1.

In the TF analysis, the TF points are rearranged from the position (ω,u) computed by the short-time Fourier transform to the position of the center of energy $\left(\omega ,\hat{u}\left(\omega ,u\right)\right)$ according to the TRST theory [25,26,27]. This rearrangement operation improves the time-frequency aggregation and accurately represents the non-stationary signal characteristics. In the far-field source localization model, distances from the sound source to different array elements of the array are different. Assuming that the first element of the array is a reference element, the time difference between the arrival of a signal at the reference element and its arrival at the other elements of the array remains constant if the direction of the signal source is unchanged. However, the time difference between the background noise received by different array elements of the array is random. According to the different nature of the time difference between the signal and noise received by the array elements, the TRST rearrangement theory is used to separate the signal TF points from the noise TF points in the short-time cross-power spectrum (STCPS). Each TF point of the STCPS is reassigned from its computed position (ω,u) to the position of the center of the gradient of the TDOA $\left(\omega ,\hat{\tau }\left(\omega ,u\right)\right)$. After the rearrangement, the TF points of the signal are clustered, whereas the TF points of the noise are still randomly distributed in the time-delay domain. The signal and noise can be separated by extracting the TF points at the center of gravity of the TDOA.

**Figure 2:**
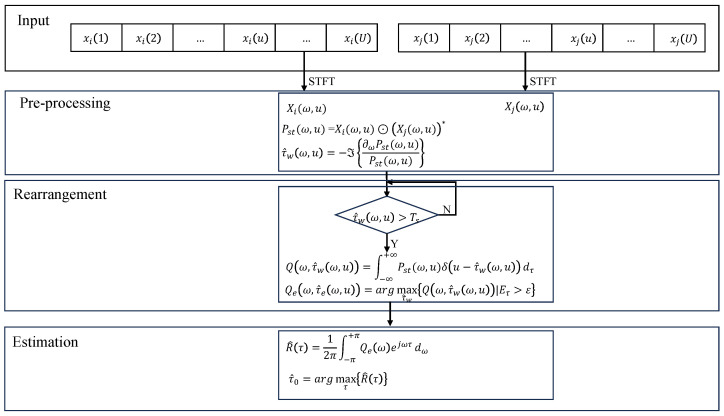
The flowchart of the proposed method.

The workflow of the proposed algorithms is presented in Figure 2, where it can be seen that it consists of three steps. First, TDOA can be estimated only by data from multiple spatially separated sensors. Second, the TF points with a stronger SNR are extracted by the rearrangement methods. Finally, the maximum value of the cross-correlation function is determined to realize a more robust estimate of the TDOA.

### 2.1. Time Difference Reassigned Synchrosqueezing Transform Algorithm

**Figure 3:**
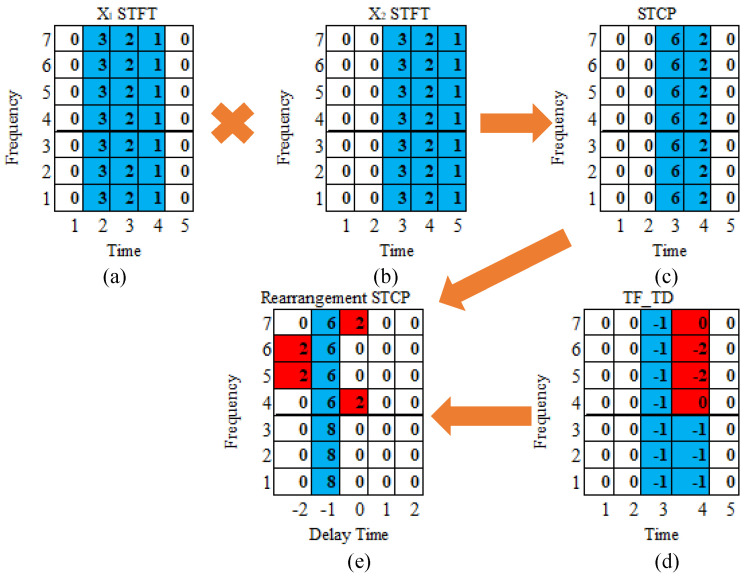
The schematic diagram of the TDST algorithm: (**a**) STFT of $x_{1}\left(t\right)$; (**b**) STFT of $x_{2}\left(t\right)$; (**c**) short-time cross-power; (**d**) the time delay of the TF points; (**e**) short-time cross-power after rearrangement.

In this study, the rearrangement method based on the TDOA is the time difference rearrangement synchrosqueezing transform (TDST) algorithm. The implementation process of the TDST algorithm is illustrated in Figure 3.

Figure 3 illustrates the process of separating the signal TF points from the noise TF points. Particularly, Figure 3c represents the STCPS, which is obtained from the dot product of the STFT of $x_{1}\left(t\right)$ and $x_{2}\left(t\right)$. Further, Figure 3d represents the time delay at each TF point in the STCPS, obtained by the derivative of the STCPS with respect to the frequency variable. The time delays of partial TF points are deviated due to the noise interference, as shown in red. Each TF point in Figure 3c is rearranged along the time-delay axis according to the time-delay in Figure 3d. As shown in Figure 3e, the TF points of the signal are aligned on the same time delay (as shown in blue), and the TF points of the noise are randomly distributed on both sides of the time delay energy ridges.

#### 2.1.1. Noise-Free TDST Model

Assume that in a noise-free environment, the transmitted signal is unknown, and the TDOA can be estimated only by data from multiple spatially separated sensors. It should be noted that the signal received by the first sensor is usually set as a reference signal.

(1) $$x_{1}\left(t\right)=a_{1}s\left(t\right)$$

(2) $$x_{2}\left(t\right)=a_{2}s\left(t-\tau_{0}\right)$$

where $x_{1}\left(t\right)$ and $x_{2}\left(t\right)$ represent the output signals of sensors #1 and #2, respectively; $a_{1}$ and $a_{2}$ denote the amplitude attenuation coefficients of the signals received by sensors #1 and #2, respectively; $\tau_{0}$ is the time difference of the signal arrival at sensor #2 relative to sensor #1.

To analyze the frequency features of a non-stationary signal, the STFT is computed by the method of a sliding window in the frequency domain, which is expressed as follows:

(3) $$\begin{array}{cc} X_{1}\left(\omega ,u\right) & =\frac{1}{2\pi }\int_{0}^{\pi }a_{1}S\left(\xi \right)G^{*}\left(\xi -\omega \right)e^{j(\xi -\omega )t}d\xi \\ \; & =A_{1}\left(\omega ,u\right)e^{-j\phi_{1}\left(\omega ,u\right)} \end{array}$$

(4) $$\begin{array}{cc} X_{2}\left(\omega ,u\right) & =\frac{1}{2\pi }\int_{0}^{\pi }a_{2}S\left(\xi \right)e^{-j\omega \tau_{0}}G^{*}\left(\xi -\omega \right)e^{j(\xi -\omega )t}d\xi \\ \; & =A_{2}\left(\omega ,u\right)e^{-j\phi_{2}\left(\omega ,u\right)} \end{array}$$

where $X_{1}\left(\omega ,u\right)$ denotes the STFT of $x_{1}\left(t\right)$; $A_{1}\left(\omega ,u\right)$ and $\phi_{1}\left(\omega ,u\right)$ are the magnitude and phase of $X_{1}\left(\omega ,u\right)$, respectively; the amplitude and phase of $X_{2}\left(\omega ,u\right)$ can be obtained from $A_{2}\left(\omega ,u\right)$ and $\phi_{2}\left(\omega ,u\right)$, respectively; (3) and (4) can be used to derive the STCPS of the signal.

(5) $$\begin{array}{cc} P_{st}\left(\omega ,u\right) & =X_{1}\left(\omega ,u\right)X_{2}^{*}\left(\omega ,u\right)\\ \; & =A_{1}\left(\omega ,u\right)e^{-j\phi_{1}\left(\omega ,u\right)}{\left(A_{2}\left(\omega ,u\right)e^{-j\phi_{2}\left(\omega ,u\right)}\right)}^{*} \end{array}$$

where ${\left(.\right)}^{*}$ represents the conjugate operation, ξ denotes the frequency shift variable, and G(ω) is the frequency domain form of g(t).

To obtain time information from the phase data, the partial derivative of (6) with respect to the frequency variable ω can be used, which is expressed as follows [28]

(6) $$\begin{array}{cc} \partial_{\omega }P_{st}\left(\omega ,u\right) & =\partial_{\omega }\left\{A_{1}\left(\omega ,u\right)e^{-j\phi_{1}\left(\omega ,u\right)}{\left(A_{2}\left(\omega ,u\right)e^{-j\phi_{2}\left(\omega ,u\right)}\right)}^{*}\right\} \end{array}$$

The STFT with the window function $\frac{dG(\omega )}{d\omega }$ is denoted as $X_{1}^{G^{\prime }}\left(\omega ,u\right)$. The time-delay operator can be represented by

(7) $$\begin{array}{cc} \; & \hat{\tau }\left(\omega ,u\right)=-ℑ\left\{\frac{\partial_{\omega }P_{st}\left(\omega ,u\right)}{P_{st}\left(\omega ,u\right)}\right\}\\ \; & =-ℑ\left\{j\partial_{\omega }\phi_{2}\left(\omega ,u\right)-j\partial_{\omega }\phi_{1}\left(\omega ,u\right)\right\} \end{array}$$

where $\hat{\tau }\left(\omega ,u\right)$ is the time delay operator that contains the time delay of all TF points of a signal; since the TF points of the signal have the same time delay, the time delay operator also represents the TDOA estimation.

In a noisy environment, the time delay operator contains the time delay data on the TF points of both the signal and the noise, so the time delay operator cannot represent the TDOA estimation of the signal. However, it is possible to separate the signal’s TF points from the noise’s TF points by using the time-delay operator. A precise TDOA estimation can be obtained by extracting the signal’s TF points.

#### 2.1.2. Noise-Optimized TDST Model

Under the noisy settings, the signals received by the two sensors can be modeled as follows:

(8) $$x_{1}\left(t\right)=a_{1}s\left(t\right)+w_{1}\left(t\right)$$

(9) $$x_{2}\left(t\right)=a_{2}s\left(t-\tau_{0}\right)+w_{2}\left(t\right)$$

where s(t) is the unknown source signal, and $w_{1}\left(t\right)$ and $w_{2}\left(t\right)$ are the additive noise terms assumed to be uncorrelated with s(t).

The STFT can be calculated by

(10) $$\begin{array}{c} X_{1}\left(\omega ,u\right)=A_{1}\left(\omega ,u\right)e^{-j\phi_{1}\left(\omega ,u\right)}+W_{1}\left(\omega ,u\right)e^{-j\phi_{w1}\left(\omega ,u\right)} \end{array}$$

(11) $$\begin{array}{c} X_{2}\left(\omega ,u\right)=A_{2}\left(\omega ,u\right)e^{-j\phi_{2}\left(\omega ,u\right)}+W_{2}\left(\omega ,u\right)e^{-j\phi_{w2}\left(\omega ,u\right)} \end{array}$$

where $X_{1}\left(\omega ,u\right)$ is the STFT of $x_{1}\left(t\right)$; $A_{1}\left(\omega ,u\right)$ and $\phi_{1}\left(\omega ,u\right)$ are the amplitude and phase of $X_{1}\left(\omega ,u\right)$, respectively; $W_{1}\left(\omega ,u\right)$ is the STFT of the $n_{1}\left(t\right)$.

Next, the STCPS can be obtained by

(12) $$\begin{array}{cc} \; & P_{stn}\left(\omega ,u\right)=X_{1}\left(\omega ,u\right)X_{2}^{*}\left(\omega ,u\right)\\ \; & =\left(A_{1}\left(\omega ,u\right)e^{-j\phi_{1}\left(\omega ,u\right)}+W_{1}\left(\omega ,u\right)e^{-j\phi_{w1}\left(\omega ,u\right)}\right)\\ \; & {\left(A_{2}\left(\omega ,u\right)e^{-j\phi_{2}\left(\omega ,u\right)}+W_{2}\left(\omega ,u\right)e^{-j\phi_{w2}\left(\omega ,u\right)}\right)}^{*} \end{array}$$

where $P_{stn}\left(\omega ,u\right)$ represents the STCPS, which includes the two sensor signals’ STCPS and noise STCPS.

The partial derivative of $P_{stn}\left(\omega ,u\right)$ with respect to the frequency variable ω is expressed as follows

(13) $$\begin{array}{cc} \; & \partial_{\omega }P_{stn}\left(\omega ,u\right)=\partial_{\omega }\left\{X_{1}\left(\omega ,u\right)X_{2}^{*}\left(\omega ,u\right)\right\} \end{array}$$

The time delay operator, including the noise, can be calculated by

(14) $$\begin{array}{c} {\hat{\tau }}_{w}\left(\omega ,u\right)=-ℑ\left\{\frac{\partial_{\omega }P_{stn}\left(\omega ,u\right)}{P_{stn}\left(\omega ,u\right)}\right\} \end{array}$$

where ${\hat{\tau }}_{w}\left(\omega ,u\right)$ denotes the time delay operator, including the noise interference.

Thus, an accurate TDOA estimation cannot be achieved directly from the time delay operator.

The signal’s TF points originating from the same directions exhibit identical lags, while the noise’s TF points show random time delays. The rearrangement method can separate the signal from the noise and enhance the SNR value.

(15) $$\begin{array}{c} Q\left(\omega ,{\hat{\tau }}_{w}\left(\omega ,u\right)\right)=\int_{-\infty }^{+\infty }P_{stn}\left(\omega ,u\right)\delta \left(u-{\hat{\tau }}_{n}\left(\omega ,u\right)\right)d\tau \end{array}$$

where $Q(\omega ,{\hat{t}}_{w}\left(\omega ,u\right))$ denotes the reassignment TF spectrum.

Equation (15) rearranges the TF points from the position (ω,u) to the position $\left(\omega ,{\hat{\tau }}_{w}\left(\omega ,u\right)\right)$. Further, to improve the robustness of the TDOA estimation, the TF points on each side of the signal time-delay energy ridge are extracted as follows [29,30,31,32].

(16) $$Q_{e}\left(\omega ,{\hat{\tau }}_{e}\left(\omega ,u\right)\right)=\arg\max_{E_{\tau }>\varepsilon }Q\left(\omega ,{\hat{\tau }}_{w}\left(\omega ,u\right)\right)$$

where $Q_{e}\left(\omega ,{\hat{\tau }}_{e}\left(\omega ,u\right)\right)$ denotes a TF point whose TF energy is greater than the energy threshold at time delays; $E_{\tau }=\int_{-\pi }^{\pi }Q(\omega ,{\hat{\tau }}_{w}\left(\omega ,u\right))d\omega$ represents the energies at different time delays; ε is the energy threshold. How to choose the value of ε may be an important issue. The value of ε may depend on the real situation or physical environment. If the value of ε is chosen improperly, numerical simulations may yield inaccurate or unreasonable results.

Further, we extract the TF points on each side of the time-delay energy ridge. A one-dimensional frequency distribution is obtained by accumulating the TF points at different time delays along the time-delay axis as follows

(17) $$Q_{e}\left(\omega \right)=\int_{-\tau }^{\tau }Q_{e}\left(\omega ,{\hat{\tau }}_{e}\left(\omega ,u\right)\right)d\tau$$

where $Q_{e}\left(\omega \right)$ denotes the frequency on both sides of the time-delay energy ridge.

The cross-correlation (CC) function constructed from $Q_{e}\left(\omega \right)$ can be derived as follows

(18) $$\hat{R}\left(\tau \right)=\frac{1}{2\pi }\int_{-\pi }^{\pi }Q_{e}\left(\omega \right)e^{j\omega \tau }d\omega$$

where $\hat{R}\left(\tau \right)$ represents the CC function.

The time delay corresponding to the peak of the CC function indicates the TDOA estimation, which is given by

(19) $${\hat{\tau }}_{0}=\arg\max_{\tau }\left(\hat{R}\left(\tau \right)\right)$$

where ${\hat{\tau }}_{0}$ denotes the TDOA estimation.

The accuracy and robustness of the delay estimation can be improved by performing the rearrangement and extraction operations. The discrete form of the algorithm is given in Algorithm 1.

**Algorithm 1:** The time difference reassigned synchrosqueezing transform algorithm.
**Initialization: $\gamma ,\beta ,Q\left[N,2N\right],\hat{t}\left[N,N\right],{\hat{\tau }}_{w},\tau_{0}$**
Input: $x_{1}\left[n\right],x_{2}\left[n\right]$Calculate: $X_{1}^{G},X_{2}^{G},X_{1}^{G^{\prime }},X_{2}^{G^{\prime }}$,  based on (10)for k ← 1 to *N*   for b ← 1 to *N*      if $\left|X_{1}^{G}{\left(X_{2}^{G}\right)}^{*}\right|>\gamma$ then         $\hat{t}\left[k,b\right]\leftarrow -ℑ\left\{\frac{-X_{1}^{G^{\prime }}{\left(X_{2}^{G}\right)}^{*}-X_{1}^{G}{\left(X_{2}^{G^{\prime }}\right)}^{*}}{X_{1}^{G}{\left(X_{2}^{G}\right)}^{*}}\right\}$,  based on  (14)      else $\hat{t}\left[k,b\right]\leftarrow 0$      end if   end forend forfor k ← 1 to *N*   for b ← -*N* to (*N*-1)      if $\hat{t}\left[k,b\right]>\beta$         ${\hat{\tau }}_{w}\leftarrow \min\left\{\max\left\{round\left(\frac{\hat{t}\left[k,b\right]}{T_{s}}\right),-N\right\},\;N-1\right\}$         $Q\left[k,{\hat{\tau }}_{w}\right]\leftarrow Q\left[k,{\hat{\tau }}_{w}\right]+X_{1}^{G}{\left(X_{2}^{G}\right)}^{*}\left[k,b\right]$,  based on (15)      end if   end forend for$Q_{e}\left[k,{\hat{\tau }}_{e}\right]=\arg\max_{E_{\tau }>\epsilon }\left(Q[k,{\hat{\tau }}_{w}]\right)$,  based on (16)$Q_{e}\left[k\right]=\sum_{{\hat{\tau }}_{e}=-N}^{N-1}Q_{e}\left[k,{\hat{\tau }}_{e}\right]$, based on  (17)$\hat{R}\left[\tau \right]\leftarrow \frac{1}{N}\sum_{k=1}^{N}Q\left(k\right)e^{\frac{j2\pi nk}{N}}$,  based on (18)${\hat{\tau }}_{0}=\arg\max_{\tau }\left(\hat{R}\left[\tau \right]\right)$,  based on (19)

### 2.2. Second Order-Based Time-Delay Reassignment Synchrosqueezing Transform Model

Based on the idea of improving the TF aggregation result to enhance the performance of the TDOA estimation, an S-TDST algorithm is proposed. In the S-TDST method, each value of the TF points is shifted from its original position at (ω,u) to the new position of $\left(\omega ,{\hat{\tau }}^{s}\left(\omega ,u\right)\right)$. The first-order Taylor expansion of phase and second-order time-delay operator are combined to achieve this. The second-order time-delay operator is given by [33,34]

(20) $${\hat{\tau }}^{s}\left(\omega ,u\right)=\left\{\begin{array}{c} \hat{\tau }\left(\omega ,u\right)-D\left(\omega ,u\right)\left(\omega -\xi \left(\omega ,u\right)\right),\\ \;\partial_{\omega }\xi \left(\omega ,u\right)\neq 0\\ \hat{\tau }\left(\omega ,u\right),\;others \end{array}\right.$$

where ${\hat{\tau }}^{s}\left(\omega ,u\right)$ denotes the second-order time-delay operator; $\hat{\tau }\left(\omega ,u\right)$ is the time delay operator; D(ω,u) represents the gradient operator [35,36], and it is defined by

(21) $$D\left(\omega ,u\right)=\frac{\partial_{\omega }\hat{\tau }\left(\omega ,u\right)}{\partial_{\omega }\hat{\xi }\left(\omega ,u\right)}$$

where $\partial_{\omega }\hat{\xi }\left(\omega ,u\right)$ represents the frequency operator’s partial derivative concerning ω.

Further, $\partial_{\omega }\hat{\tau }\left(\omega ,u\right)$ refers to the partial derivative of the time delay operator in relation to ω.

(22) $$\begin{array}{cc} \partial_{\omega }\hat{\tau }\left(\omega ,u\right) & =-\partial_{\omega }\left\{ℑ\left\{\frac{\partial_{\omega }P_{stn}\left(\omega ,u\right)}{P_{stn}\left(\omega ,u\right)}\right\}\right\}\\ \; & =\frac{jX_{1}^{G}X_{1}^{G^{"}}{\left(X_{2}^{G}\right)}^{2}-j{\left(X_{1}^{G}\right)}^{2}{\left(X_{2}^{G}\right)}^{*}{\left(X_{2}^{G^{"}}\right)}^{*}}{{\left(X_{1}^{G}{\left(X_{2}^{G}\right)}^{*}\right)}^{2}}\\ \; & +\frac{j{\left(X_{1}^{G^{{}^{\prime }}}\right)}^{2}{\left(X_{2}^{G}\right)}^{2}+j{\left(X_{1}^{G}\right)}^{2}{\left(X_{2}^{G^{{}^{\prime }}}\right)}^{2}}{{\left(X_{1}^{G}{\left(X_{2}^{G}\right)}^{*}\right)}^{2}} \end{array}$$

where $X_{1}^{G}$ is the STFT calculated using the window function G(ω) and signal $x_{1}\left(t\right)$; $X_{1}^{G^{\prime }}$ is the STFT obtained from the window function $\frac{dG(\omega )}{d_{\omega }}$ and signal $x_{1}\left(t\right)$; $X_{1}^{G^{\prime \prime }}$ is used to calculate the STFT by using the product of the second derivative of the window function $\frac{d^{2}G\left(\omega \right)}{d_{\omega }}$ and signal $x_{1}\left(t\right)$.

The value of $\partial_{\omega }\hat{\xi }\left(\omega ,u\right)$ can be calculated by [33,34]

(23) $$\begin{array}{cc} \partial_{\omega }\xi \left(\omega ,u\right) & =\partial_{\omega }\left\{ℑ\left\{\frac{\partial_{u}P_{stn}\left(\omega ,u\right)}{P_{stn}\left(\omega ,u\right)}\right\}\right\}\\ \; & =\frac{-X_{1}^{G}X_{1}^{\omega G^{{}^{\prime }}}{\left(X_{2}^{G}\right)}^{2}+{\left(X_{1}^{G}\right)}^{2}{\left(X_{2}^{G}\right)}^{*}{\left(X_{2}^{\omega G^{{}^{\prime }}}\right)}^{*}}{{\left(X_{1}^{G}{\left(X_{2}^{G}\right)}^{*}\right)}^{2}}\\ \; & +\frac{X_{1}^{G^{{}^{\prime }}}X_{1}^{\omega G}{\left(X_{2}^{G}\right)}^{2}-{\left(X_{1}^{G}\right)}^{2}{\left(X_{2}^{G^{{}^{\prime }}}\right)}^{*}{\left(X_{2}^{\omega G}\right)}^{*}}{{\left(X_{1}^{G}{\left(X_{2}^{G}\right)}^{*}\right)}^{2}} \end{array}$$

where $X_{1}^{\omega G^{{}^{\prime }}}$ is the STFT obtained using the window function $\frac{\omega dG(\omega )}{d_{\omega }}$ and signal $x_{1}\left(u\right)$; $X_{1}^{\omega G}$ is the STFT calculated by the window function ωG(ω) and signal $x_{1}\left(t\right)$.

The gradient operator in (21) can be further derived as follows [37]

(24) $${\hat{\tau }}_{n}^{s}\left(\omega ,u\right)=\left\{\begin{array}{cc} \; & \frac{-jX_{1}^{G^{{}^{\prime }}}{\left(X_{2}^{G}\right)}^{*}-jX_{1}^{G}{\left(X_{2}^{G^{{}^{\prime }}}\right)}^{*}}{X_{1}^{G}{\left(X_{2}^{G}\right)}^{*}}\\ \; & -D\left(\omega ,u\right)\left(\omega -\frac{X_{1}^{\omega G}{\left(X_{2}^{G}\right)}^{*}-X_{1}^{G}{\left(X_{2}\omega G\right)}^{*}}{X_{1}^{G}{\left(X_{2}^{G}\right)}^{*}}\right),\\ \; & \;X_{1}^{G}{\left(X_{2}^{G}\right)}^{*}\neq 0\\ \; & \frac{-jX_{1}^{G^{{}^{\prime }}}{\left(X_{2}^{G}\right)}^{*}-jX_{1}^{G}{\left(X_{2}^{G^{{}^{\prime }}}\right)}^{*}}{X_{1}^{G}{\left(X_{2}^{G}\right)}^{*}},\;others \end{array}\right.$$

According to (24), the second-order time-delay operator ${\hat{\tau }}^{s}\left(\omega ,u\right)$ can accurately describe the delay time estimation result. To separate the signal’s TF points from the noise’s TF points, a rearranging process can be adopted as follows

(25) $$Q^{s}\left(\omega ,{\hat{\tau }}_{n}^{s}\left(\omega ,u\right)\right)=\int_{-\infty }^{+\infty }P_{stn}\left(\omega ,u\right)\delta \left(u-{\hat{\tau }}_{n}^{s}\left(\omega ,u\right)\right)d\tau$$

where $Q^{s}\left(\omega ,{\hat{\tau }}_{n}^{s}\left(\omega ,u\right)\right)$ denotes the rearranged TF spectrum.

Equation (25) shows that the TF points are rearranged from position (ω,u) to the position $\left(\omega ,{\hat{\tau }}_{n}^{s}\left(\omega ,u\right)\right)$. To improve the SNR, the TF points on each side of the signal time-delay energy ridge are extracted.

(26) $$Q_{e}^{s}\left(\omega ,{\hat{\tau }}_{e}^{s}\left(\omega ,u\right)\right)=\arg\max_{E_{\tau }>\varepsilon }Q\left(\omega ,{\hat{\tau }}_{n}^{s}\left(\omega ,u\right)\right)$$

where $Q_{e}^{s}\left(\omega ,{\hat{\tau }}_{e}^{s}\left(\omega ,u\right)\right)$ denotes the TF point where the TF energy is larger than the energy threshold.

Next, the TF points on each side of the time-delay energy ridge are extracted. A one-dimensional frequency distribution is obtained by accumulating the TF points at different time delays along the time-delay axis.

(27) $$Q_{e}^{s}\left(\omega \right)=\int_{-\tau }^{\tau }Q_{e}^{s}\left(\omega ,{\hat{\tau }}_{e}^{s}\left(\omega ,u\right)\right)d\tau$$

where $Q_{e}^{s}\left(\omega \right)$ is the cross-power spectrum.

The CC function constructed from $Q_{e}^{s}\left(\omega \right)$ can be derived as follows

(28) $${\hat{R}}_{s}\left(\tau \right)=\frac{1}{2\pi }\int_{-\pi }^{\pi }Q_{e}^{s}\left(\omega \right)e^{j\omega \tau }d\omega$$

where ${\hat{R}}_{s}\left(\tau \right)$ represents the CC function.

The time delay corresponding to the peak of the CC function denotes the TDOA estimation result, which is given by

(29) $${\hat{\tau }}_{0}^{s}=\arg\max_{\tau }{\hat{R}}_{s}\left(\tau \right)$$

where ${\hat{\tau }}_{0}^{s}$ denotes the TDOA estimation result.

The TF points of the signal are separated from the TF points of the noise based on their different time-delay properties. Since the TF points of the signal have the same time delay while the time delay of the noise is random, the time-delay energy ridges of the signal are formed during the rearrangement process. The SNR is improved by extracting the TF points on the energy ridge and both of its sides, which enhances the accuracy and robustness of delay estimation. Algorithm 2 outlines the specific steps of the procedure.

**Algorithm 2:** The second order-based time difference reassigned synchrosqueezing transform algorithm.
**Initialization: $\gamma ,Q\left[2N,2N\right],\hat{t}\left[2N,2N\right],\hat{\tau }\left[2N\right],\tau_{0}$**
Input: $x_{1}\left(t\right),x_{2}\left(t\right)$Calculate: $X_{1}^{G},X_{2}^{G},X_{1}^{G^{\prime }},X_{2}^{G^{\prime }},X_{1}^{G^{{}^{\prime \prime }}},X_{2}^{G^{{}^{\prime \prime }}},X_{1}^{\omega G^{{}^{\prime }}},X_{2}^{\omega G^{{}^{\prime }}},X_{1}^{\omega G},X_{2}^{\omega G}$for l ← 1 to 2*N*   for b ← 1 to *N*      if $\left|X_{1}^{G}{\left(X_{2}^{G}\right)}^{*}\right|>\gamma$ then         ${\hat{\tau }}^{s}\left[l,b\right]\leftarrow -ℑ\left\{\frac{-X_{1}^{G^{\prime }}{\left(X_{2}^{G}\right)}^{*}-X_{1}^{G}{\left(X_{2}^{G^{\prime }}\right)}^{*}}{X_{1}^{G}{\left(X_{2}^{G}\right)}^{*}}\right\}-D\left(\omega ,t\right)\left(\omega -\xi \left(\omega ,t\right)\right)$    based on (20)      else ${\hat{\tau }}^{s}\left[l,b\right]\leftarrow -ℑ\left\{\frac{-X_{1}^{G^{\prime }}{\left(X_{2}^{G}\right)}^{*}-X_{1}^{G}{\left(X_{2}^{G^{\prime }}\right)}^{*}}{X_{1}^{G}{\left(X_{2}^{G}\right)}^{*}}\right\}$      end if   end forend forfor l ← 1 to 2*N*   for b ← -*N* to (*N*-1)      if $\hat{\tau }\left[l,b\right]>\beta$         $s\leftarrow \min\left\{\max\left\{round\left(\frac{{\hat{\tau }}^{s}\left[l,b\right]}{T_{s}}\right),-N\right\},N-1\right\}$         $Q^{s}\left[l,s\right]\leftarrow Q^{s}\left[l,s\right]+X_{1}^{G}{\left(X_{2}^{G}\right)}^{*}\left[l,b\right]$    based on (25)      end if   end forend for${\hat{R}}_{g}\left(\tau \right)\leftarrow \frac{1}{2\pi }\int_{-\pi }^{\pi }Q^{s}\left(\omega \right)e^{j\omega \tau }d\omega$,   based on (28)${\hat{\tau }}_{0}^{s}=\arg\max_{\tau }\left({\hat{R}}_{s}\left(\tau \right)\right)$   based on (29)

## 3. Simulation and Experimental Results

The effectiveness of the proposed algorithms was first verified by using the simulation data, and then their robustness and accuracy were verified by using real data. Furthermore, the proposed algorithms were compared with the related algorithms, including the generalized cross-correlation (GCC) transform, singular value decomposition frequency-sliding generalized cross-correlation (GCCsvd), and weight singular value decomposition frequency-sliding generalized cross-correlation (GCCwsvd) [18]. The proposed algorithms’ evaluation parameters included the Gaussian window with a window length of $\sigma =1.2\times 10^{-4}$ and a window overlap of 75%, using 512 points for Fast Fourier Transform. The FS-GCC algorithm used a Hanning window with a length of 32 points, a 75% overlap, and a Fast Fourier Transform of 512 points for optimal performance. An accurate estimation of the time delay was defined as follows

(30) $$\left|{\hat{\tau }}_{i}-\tau_{0}\right|\leq 1\;sampling\;points,\;i=1,\ldots M$$

where ${\hat{\tau }}_{i}$ is the estimated time delay of the *i*th Monte Carlo simulation, and *M* is the number of simulation runs, and it was set to 500 in this study.

Moreover, the root mean square error (RMSE) was used to evaluate the performance of the time delay estimation of different algorithms, and it was calculated as follows

(31) $$RMSE=\sqrt{\frac{1}{M}\sum_{i=1}^{M}{\left({\hat{\tau }}_{i}-\tau_{0}\right)}^{2}}$$

where $\tau_{0}$ denotes the actual time delay.

### 3.1. Simulation Results and Analysis

In the simulations, it was assumed that the transmitting signal was an impulsive signal. The sampling frequency was set to *Fs* = 32 kHz (i.e., the sampling interval was $T_{s}=1/Fs$), the signal length was 2 ms, and the time delay was $\tau_{0}=-3T_{s}$. The attenuation coefficient of the signal reaching the second sensor was 85% of the signal reaching the first sensor. The source signal s(t) was defined in the frequency domain as follows

(32) $$S\left(\omega \right)=S_{1}\left(\omega \right)+S_{2}\left(\omega \right)+S_{3}\left(\omega \right)$$

where,

(33) $$\begin{array}{c} S_{1}\left(\omega \right)=0.5e^{-j0.1\pi \omega } \end{array}$$

(34) $$\begin{array}{c} S_{2}\left(\omega \right)=e^{-j(\omega -0.05\omega^{2}+0.006\omega^{3})} \end{array}$$

(35) $$\begin{array}{c} S_{3}\left(\omega \right)=e^{-j(0.6\omega -2e^{0.2-0.03\omega }.*\sin\left(0.2\omega \right))} \end{array}$$

**Figure 4:**
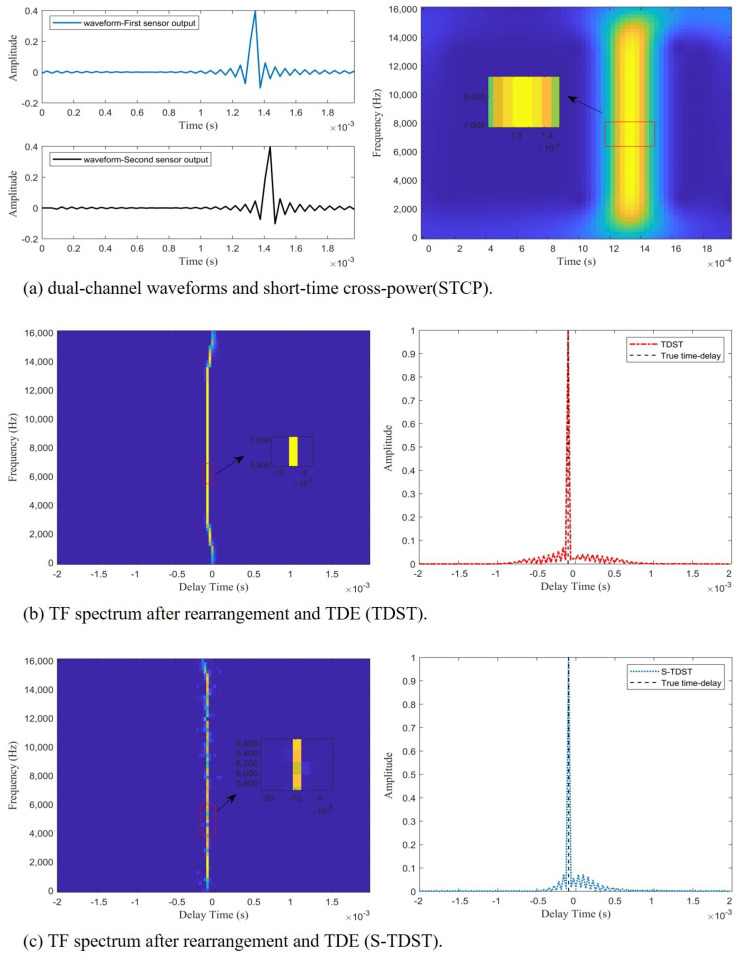
The time delay estimation process of the proposed algorithms; the red curve indicates the TDST, and the blue curve denotes the S-TDST.

Experiment 1. This experiment was conducted to verify the effectiveness of the proposed algorithms in a noise-free environment. Figure 4 displays the TDE process implemented by the proposed algorithms.

Figure 4 demonstrates the effectiveness of the proposed algorithms. As presented on the left side of Figure 4b,c, a straight line is approximately perpendicular to the time-delay axis, which indicates that the proposed algorithms could rearrange the TF points according to the time-delay property. This straight line is called the time-delay energy ridge. The peaks of the red and blue curves coincide with the black dashed line, as displayed on the right side of Figure 4b,c, which demonstrates that the proposed algorithms could accurately estimate time delay based on the time-delay energy ridges.

**Figure 5:**
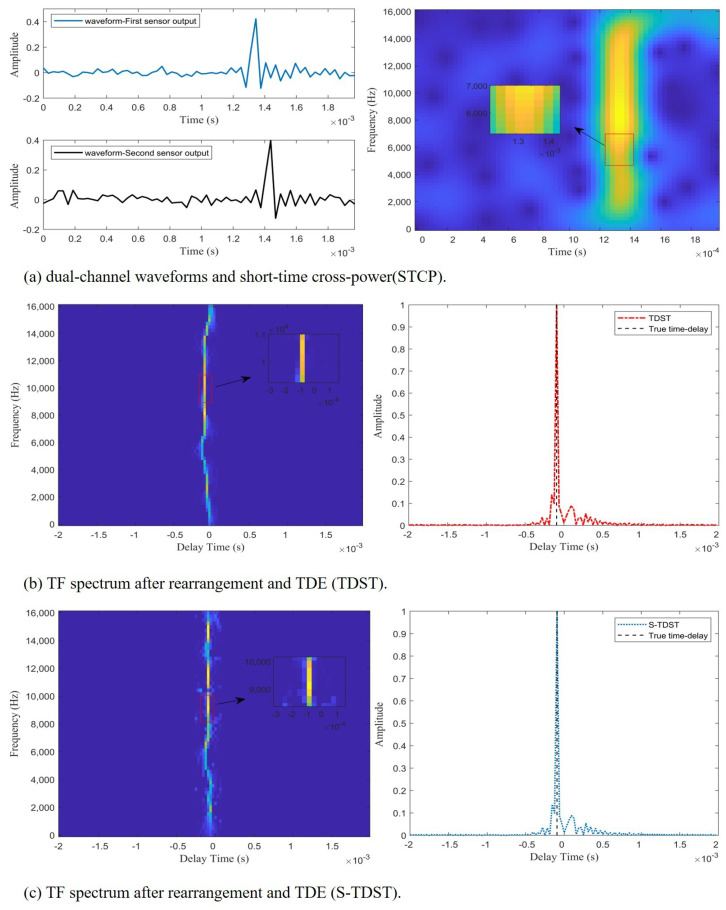
The TDE performance of the proposed algorithms in a noisy environment at SNR = 10 dB.

Experiment 2. This experiment was performed to verify the effectiveness of the proposed algorithms in a noisy environment. The performance of the proposed methods in a noisy environment is shown in Figure 5.

Figure 5 illustrates the effectiveness and accuracy of the proposed approaches in estimating the time delay in noisy environments. As shown on the right side of Figure 5b,c, the peaks of the red and blue curves coincide with the black dashed line, which indicates that the proposed algorithms could accurately estimate the time delay in noisy environments. Figure 5a displays the impulsive signals’ waveforms of the dual channels and STCPS. Figure 5b shows the time-delay energy ridges extracted by the TDST and their TDE spectra. The time-delay energy ridges and TDE spectrum obtained by the S-TDST method are depicted in Figure 5c. Comparing the results presented on the right side of Figure 5a and Figure 4a, it can be concluded that many TF points were disturbed by the noise, but some TF points were still unaffected. This demonstrated why the algorithm can accurately estimate the time delay in a noisy environment. Comparing the results displayed on the left side of Figure 5b and Figure 4b, it can be concluded that the TF points unaffected by noise were observed to be distributed along the time-delay energy ridge. In contrast, the TF points interfered with by the noise were randomly distributed on both sides of the time-delay energy ridge. Figure 5 illustrates that extracting the TF points on the time-delay energy ridges that were not disturbed by the noise could enable TDOA estimation.

### 3.2. Experiments under Real-Life Settings

**Figure 6:**
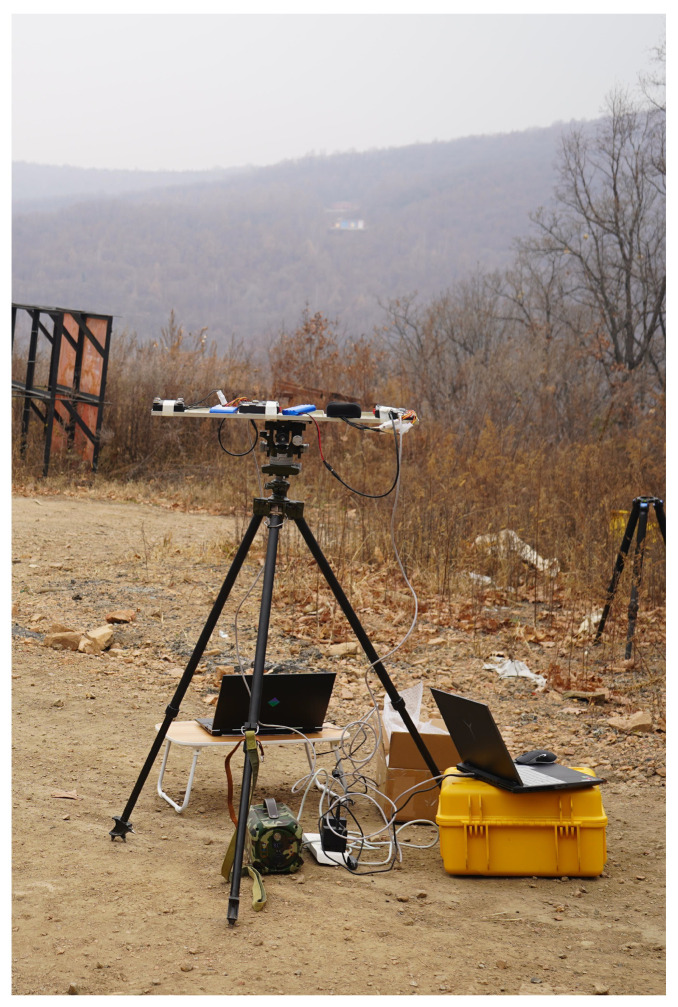
Experimental site.

The experiment was conducted to acquire the shock wave signal, as illustrated in Figure 6.

Figure 6 shows the test equipment for acquiring shock waves in an outdoor far-field environment. In Figure 6, the second black square to the right above the tripod represents an embedded quadrature planar microphone array system used to capture and measure the shock waves. In Figure 6, directly below the tray is the instrumentation, which was used to calibrate the distance and bearing of the firing point. The embedded quadrature planar microphone array system was fixed horizontally on a tripod, and the target azimuth was equivalently changed from small to large by rotating the array system horizontally clockwise, with the center of the array as the coordinate origin. The firing points and targets were at the same height as the microphone array. In these experiments, the target azimuth was increased by a 15-degree step from zero, and 20 independent trials were conducted at each target azimuth value. Further, there was no obstruction between the microphone array and the firing point location, and the distance between them was set to 1000 m. Two array elements were randomly selected from the array, and the shock wave data that were collected by both elements were acquired. The shock wave data were digitized to 16 bits with a sampling rate of *Fs* = 32 kHz. The proposed algorithm’s evaluation parameters include a Gaussian window with a window length of $\sigma =1.2\times 10^{-4}$ and a window overlap of 75%, using 512 points for Fast Fourier Transform. The FS-GCC algorithm used a Hanning window with a length of 32 points, a 75% overlap, and a Fast Fourier Transform of 512 points for optimal performance.

**Figure 7:**
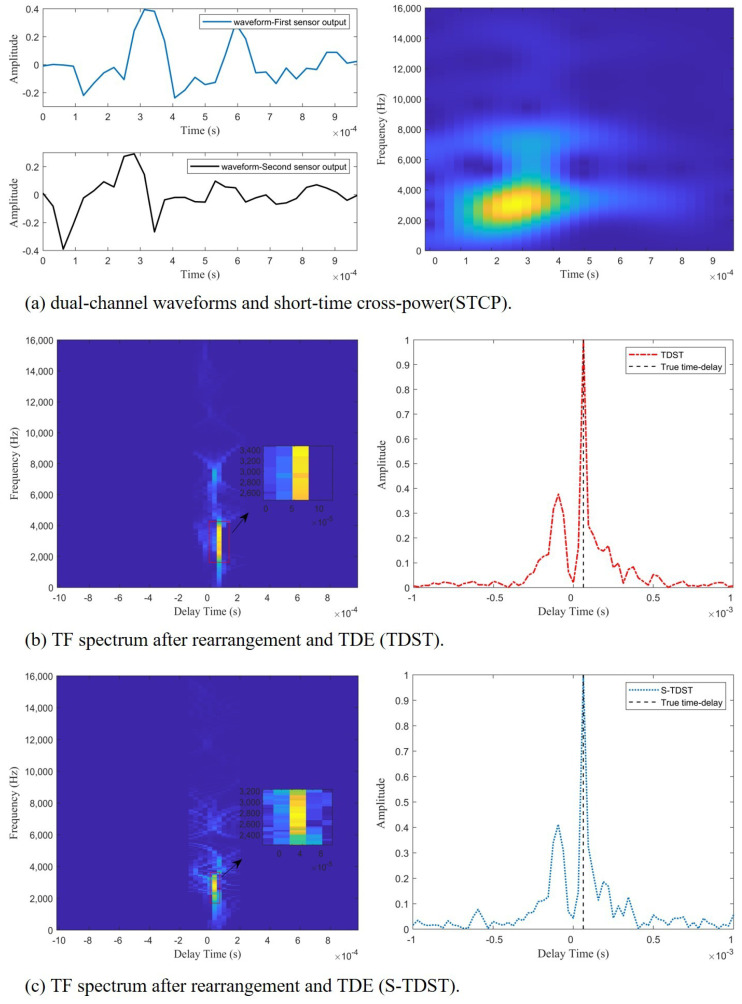
The performances of the proposed algorithms in the TDE of the shock wave signals at SNR = 10 dB.

Experiment 3. This experiment was performed to verify the effectiveness of the proposed algorithms in the TDE of shock wave signals. The performance of the proposed methods is presented in Figure 7.

Figure 7 demonstrates the effectiveness of the proposed algorithm in the TDE of the shock wave signals. As shown in Figure 7b,c, the black dashed line aligns with the highest points of the red and blue curves, which indicates that the proposed algorithms could accurately estimate the time delay.

Figure 7a displays the double channel’s shock waveform and the STCPS; Figure 7b depicts the STCPS of the rearrangement process and the TDE spectrum obtained by the TDST; Figure 7c displays the STCPS of the rearrangement process and the TDE spectrum obtained by the S-TDST. Comparing the results presented on the right side of Figure 7a and Figure 5a, it can be concluded that the STCPS of the muzzle blast signals was similar to the STCPS of the ideal impulsive signal in a noisy environment. The TF points of the STCPS of muzzle blast were aligned on the time-delay energy ridge, as shown on the left side of Figure 7b and the right side of Figure 7a.

Comparing the results presented on the left side of Figure 7b and Figure 5b, it can be concluded that the time-delay energy ridge of the shock waves was similar to that of the impulsive signals in the noise environment. This proved the effectiveness of the proposed algorithm in the TDE of the shock wave signals.

**Figure 8:**
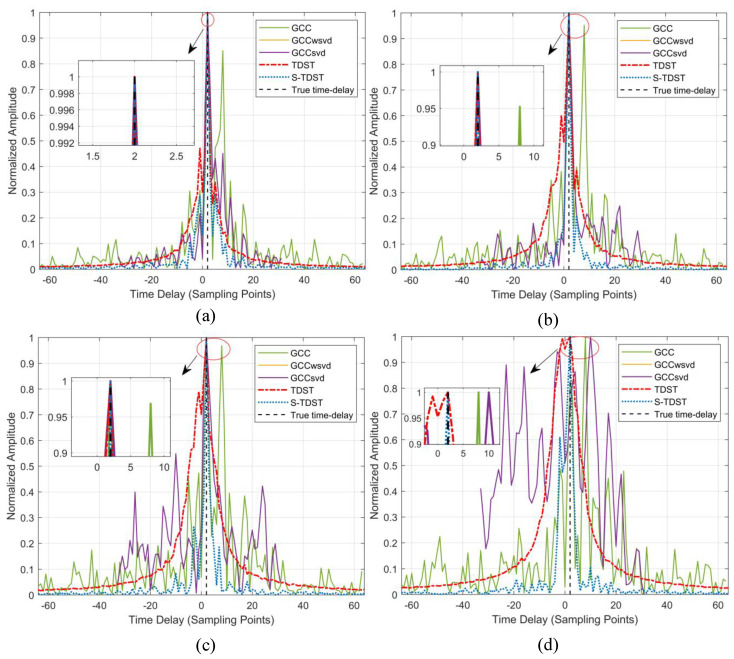
Comparison of the performance results obtained under different SNR values: (**a**) SNR of 5 dB; (**b**) SNR of 0; (**c**) SNR of −5 dB; (**d**) SNR of −10 dB.

Experiment 4. This experiment was performed to verify the proposed algorithms’ robustness in the varying SNR environments. The SRN level was set to 5 dB, 0, −5 dB, and −10 dB, in turn. Figure 8 shows the average TDE spectrum in varying SNR environments.

Figure 8 illustrates that the TDE performances of all the algorithms were enhanced under increased SNR value. Figure 8a,b show that all algorithms, except the GCC algorithm, could accurately estimate the time delay. The main lobes and sidelobes in the GCC algorithm were almost of the same height, which caused this algorithm to perform poorly. In Figure 8d, it can be seen that only the S-TDST algorithm could achieve valid delay estimates. Although the TDST algorithm could obtain accurate time delay, its sidelobe was considerably high. Further, the comparison algorithm yielded incorrect delay estimates. Figure 8 demonstrates that the performance of the proposed algorithms outperformed those of the comparison algorithms in the low-SNR environments.

**Figure 9:**
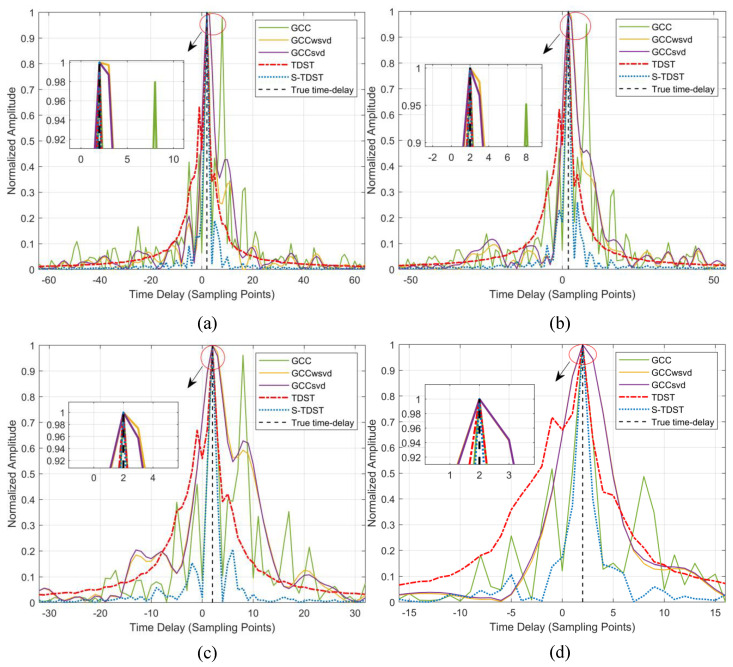
Performance results of the algorithms obtained for different signal lengths: (**a**) signal length was 64 sampling points; (**b**) signal length was 54 sampling points; (**c**) signal length was 32 sampling points; (**d**) signal length was 16 sampling points; the black dashed line denotes the real delays; SNR = 0 dB.

Experiment 5. This experiment was conducted to verify the robustness of the proposed algorithms in TDE with various signal sizes. Figure 9 displays the performance of all the algorithms obtained under varying signal lengths.

Figure 9 illustrates that both the proposed and the comparison algorithms had good adaptability to the data size variations. Figure 9a–d show that all algorithms could accurately estimate the time delay. By comparing the results presented in Figure 9a,d, it can be observed that the proposed S-TDST algorithm had the narrowest main lobe and the lowest sidelobe among all algorithms. This indicated that the robustness of the S-TDST outperformed that of the comparison algorithms.

**Figure 10:**
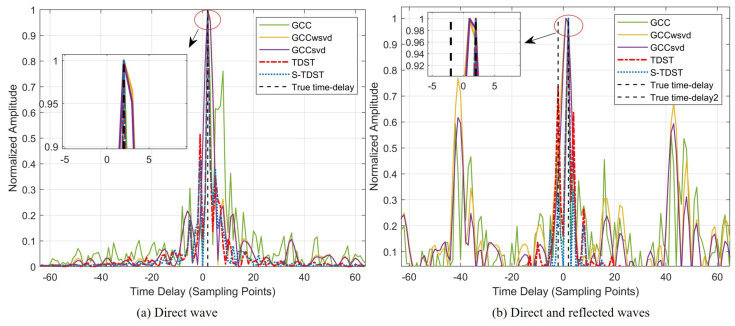
Performance comparison in the multi-path environment.

Experiment 6. This experiment was performed to verify the effectiveness of the proposed algorithms under the multi-path conditions. The SRN level was set to 20 dB. Figure 10 shows the average delay estimation spectrum versus different multi-path numbers.

Figure 10 shows that the proposed algorithms had better robustness than the comparison algorithms in the multi-path environment. Figure 10a reveals that all algorithms could accurately estimate the time delay in the multi-path-free environment. However, contrastive algorithms exhibit higher sidelobes, which reduce their robustness. Further, Figure 10b shows that the proposed algorithms could accurately estimate the time delay of both direct and multi-path signals, whereas the contrastive algorithms failed to do so.

**Figure 11:**
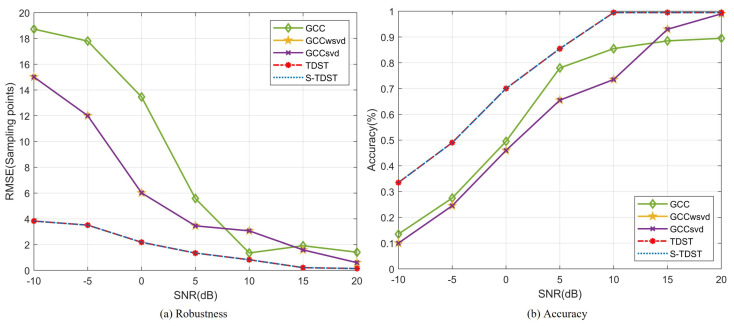
Performance comparison under different SNR values.

Experiment 7. This experiment was performed to verify the accuracy and robustness of proposed algorithms in the varying-SNR environments. Figure 11 shows the robustness and accuracy of the proposed and comparison algorithms under varying-SNR conditions.

Figure 11 shows that the robustness and accuracy of the proposed algorithms outperformed the comparison algorithms in the varying-SNR environment. In Figure 11a, the TDE robustness values of all the algorithms are displayed for varying SNR levels. The results indicated that the contrastive approach showed certain instability in low-SNR environments despite achieving better estimation stability under high-SNR settings. As SNR decreased from 5 dB to −10 dB, the red and blue curves demonstrated smooth changes, indicating excellent robustness for the proposed algorithms. However, the green curve increased steeply, indicating the poor robustness of the GCC algorithm in low-SNR environments. Figure 11b displays the accuracy performance of all algorithms with the variation in the SNR value. The results demonstrated that the proposed algorithms had good TDE accuracy, particularly when the SNR value was higher than 10 dB, with an accuracy close to 100%. Compared to the proposed algorithm, the GCCwsvd and GCCsvd algorithms had the same estimation accuracy and poorer estimation accuracy in low-SNR environments and the same accuracy in high-SNR environments. Comparing the results presented in Figure 11a,b, it can be observed that the proposed algorithms outperformed the comparison algorithms in terms of robustness and accuracy.

Experiment 8. This experiment was performed to verify the computational efficiency of the proposed algorithms and compare it with those of the comparison algorithms. Table 1 shows that the running time varied for different algorithms.

Table 1 presents the running time results of different algorithms. The computational efficiencies of the GCC, GCC-svd, and GCC-wsvd were nearly identical. However, among these algorithms, the GCC-svd had the shortest computational time. In contrast, the proposed algorithms have a long computational time; particularly, the S-TDST algorithm had the longest computational time among all algorithms. Therefore, to meet real-time computational requirements, the operational efficiency of the proposed algorithms should be improved.

The proposed algorithms exhibit higher robustness and accuracy in time delay estimation in low SNR environments compared to the comparison algorithms. However, the proposed algorithm has some areas for improvement in delay estimation. For instance, the computational efficiency of the proposed algorithms is much lower than that of the comparison algorithm, and it may not meet the real-time computational requirements. Additionally, the size of the window length in the frequency domain sliding window significantly affects the accuracy of delay estimation. Lastly, the estimation’s robustness is impacted by the bias from the numerical derivation.

## 4. Conclusions

This paper aims to improve the time delay estimation accuracy of dual-channel sensors for single-sound sources in low-SNR environments. A time difference rearrangement method suitable for low-SNR environments is proposed. This method separates the signal from the background noise by rearranging them based on their time-delay characteristics. The proposed method includes four main steps. First, a rough estimation of the time delay is obtained by calculating the partial derivative of the STCPS. Second, a rearrangement operation is performed to separate the signal’s TF points from the noise’s TF points. Third, the TF points on both sides of the time-delay energy ridge are extracted using the greedy method. Fourth, a refined TDOA estimation is realized by performing the inverse Fourier transformation on the extracted TF points. Moreover, the S-TDST method is proposed to improve the robustness of the TDOA estimation by enhancing the TF energy aggregation, thus significantly improving the estimation performance. The proposed algorithm is verified by simulations and experiments, and the results show that the proposed algorithm is more robust and accurate than the existing algorithms.

## Figures and Tables

**Table 1:** Running Time (Intel (R) Core (TM) i5-12400F CPU @2.5 GHz).

Algorithms	GCC	GCC-svd	GCC-wsvd	TDST	TDST
Time (ms)	0.3405	0.2223	0.1660	18.1	41.3

## Data Availability

Data are contained within the article.
